# Supplementary figures and images for: Tau expression and phosphorylation in enteroendocrine cells
Source: Front Neurosci. 2023 Jun 2;17:1166848. doi: 10.3389/fnins.2023.1166848 (PMC10272410; doi:10.3389/fnins.2023.1166848)

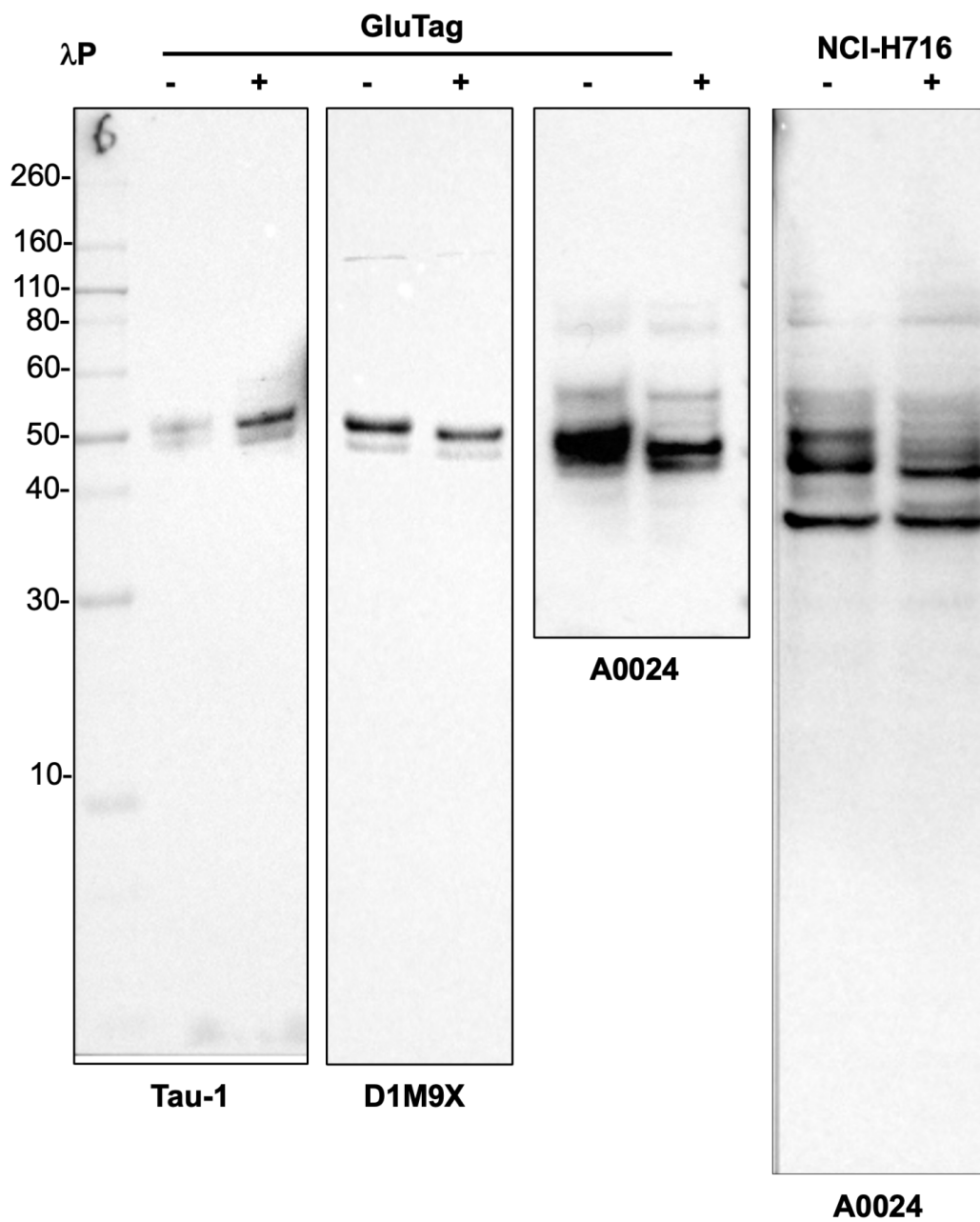

**Supplementary figure 1.** Uncropped western blots showing the absence of big tau in EEC

Supplement: Supplementary file 1 [file Data_Sheet_1.PDF]
